# High-throughput kinase assays with protein substrates using fluorescent polymer superquenching

**DOI:** 10.1186/1472-6750-5-16

**Published:** 2005-05-31

**Authors:** Frauke Rininsland, Casey Stankewicz, Wendy Weatherford, Duncan McBranch

**Affiliations:** 1 QTL Biosystems, 2778 Agua Fria Street, Santa Fe, NM 87507, USA

## Abstract

**Background:**

High-throughput screening is used by the pharmaceutical industry for identifying lead compounds that interact with targets of pharmacological interest. Because of the key role that aberrant regulation of protein phosphorylation plays in diseases such as cancer, diabetes and hypertension, kinases have become one of the main drug targets. With the exception of antibody-based assays, methods to screen for specific kinase activity are generally restricted to the use of small synthetic peptides as substrates. However, the use of natural protein substrates has the advantage that potential inhibitors can be detected that affect enzyme activity by binding to a site other than the catalytic site. We have previously reported a non-radioactive and non-antibody-based fluorescence quench assay for detection of phosphorylation or dephosphorylation using synthetic peptide substrates. The aim of this work is to develop an assay for detection of phosphorylation of chemically unmodified proteins based on this polymer superquenching platform.

**Results:**

Using a modified QTL Lightspeed™ assay, phosphorylation of native protein was quantified by the interaction of the phosphorylated proteins with metal-ion coordinating groups co-located with fluorescent polymer deposited onto microspheres. The binding of phospho-protein inhibits a dye-labeled "tracer" peptide from associating to the phosphate-binding sites present on the fluorescent microspheres. The resulting inhibition of quench generates a "turn on" assay, in which the signal correlates with the phosphorylation of the substrate. The assay was tested on three different proteins: Myelin Basic Protein (MBP), Histone H1 and Phosphorylated heat- and acid-stable protein (PHAS-1). Phosphorylation of the proteins was detected by Protein Kinase Cα (PKCα) and by the Interleukin -1 Receptor-associated Kinase 4 (IRAK4). Enzyme inhibition yielded IC_50 _values that were comparable to those obtained using peptide substrates. Statistical parameters that are used in the high-throughput community to determine assay robustness (Z'-value) demonstrate the suitability of this format for high-throughput screening applications for detection of inhibitors of enzyme activity.

**Conclusion:**

The QTL Lightspeed™ protein detection system provides a simple mix and measure "turn on" assay for the detection of kinase activity using natural protein substrates. The platform is robust and allows for identification of inhibitors of kinase activity.

## Background

**Figure 1:**
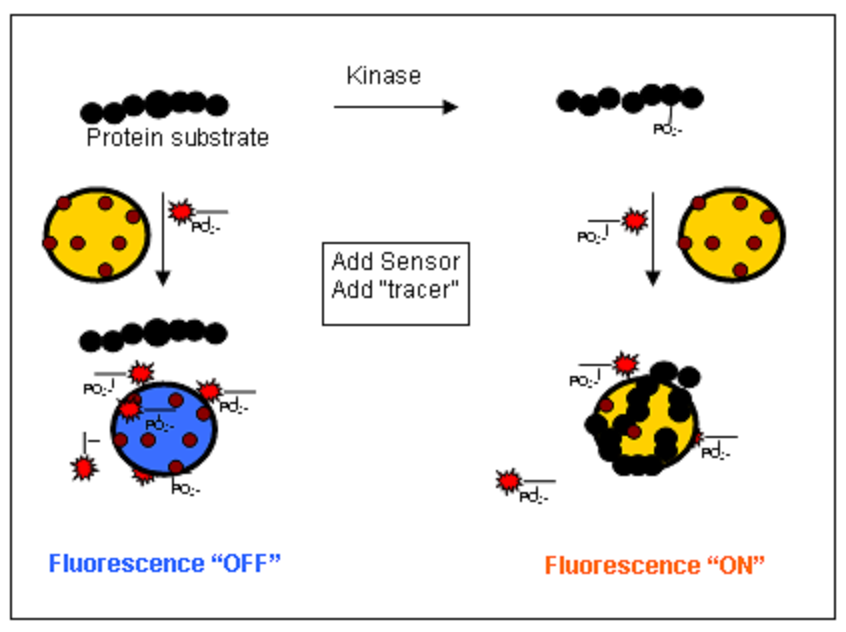
Phosphorylated protein binds to the QTL Sensor via specific phosphate binding to metal coordinating ions and inhibits the association of the dye-labeled phosphopeptide (tracer; red "starburst"). The resulting increase in fluorescence signal correlates with the extent of protein phosphorylation.

Approximately 75% of the drugs in clinical use elicit their pharmacological effects by interactions with receptor or enzyme targets, such as kinases [1,2]. Methods to screen large chemical libraries for inhibitors of protein kinases include radiometric assays [3], ELISA [4], ATP consumption assays [5] and several fluorescence based assays such as time-resolved fluorescence (TRF) [6], fluorescence polarization (FP) [7,8], fluorescence energy transfer (FRET) [9] or fluorescence quench assays [10]. Assays such as FRET, FP and fluorescence quench do not require antibodies or radioactive label, and are thus attractive for HTS. However non-antibody based FP and FRET assays are restricted to the use of small, synthetic peptide substrates to monitor kinase activity. While peptide substrates are convenient for HTS purposes, those that bind with high affinity are available for only a small percentage of the >500 kinases encoded by the human genome [11]. Additionally, peptide substrates may diminish the ability to detect inhibitors that bind to docking sites of a native protein substrate or that bind to unique conformational states induced by protein substrate binding [12]. Here we report the detection of phosphorylation of the natural protein substrates Myelin Basic Protein (MBP), Histone H1 and Phosphorylated heat- and acid-stable protein (PHAS-1) by PKCα and IRAK4 using a modified version of our original assay format [13,14], which is based on superquenching of fluorescent polyelectrolytes [15,16]. The assay principle is shown in Figure 1.

## Results

**Figure 2:**
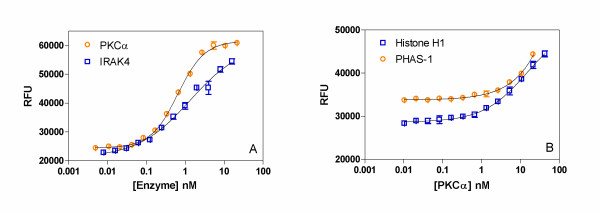
**Enzyme concentration curve using proteins as substrates**. MBP (0.5 μg/well) was phosphorylated using various amounts of PKCα and IRAK4 (2A) and Histone H1 and PHAS-1 (0.5 μg/well) were phosphorylated using various amounts of PKCα (2B) for 1 hour at room temperature (~25°C) in a 384-well white Optiplate. Following reaction, QTL Sensor was added for 10 minutes at ~25°C. Then, dye-labeled phosphopeptide tracer was added with a final concentration of 0.5 μM for detection of phosphorylation of MBP or 125 nM for Histone H1 and PHAS-1. The plate was incubated for an additional 30 minutes at ~25°C and fluorescence measured.

**Figure 3:**
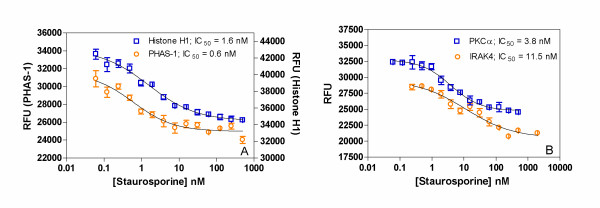
**Staurosporine inhibition of enzyme activity**. A concentration of 8.6 nM PKCα was used to phosphorylated Histone H1 or PHAS-1 for 60 minutes at ~25°C using various concentrations of Staurosporine (3A). Inhibition of PKCα and IRAK4 activities using MBP as a substrate were performed using decreasing concentrations of Staurosproine (3B). Following incubation, QTL Sensor was added and incubated for 10 minutes at ~25°C. Subsequently dye-labeled phosphopeptide tracer was added with a final concentration of 0.5 μM for MBP or 125 nM for Histone H1 and PHAS-1. The plate was incubated for an additional 30 minutes at ~25°C and fluorescence measured.

MBP, Histone H1 and and PHAS-1 proteins were phosphorylated with PKCα and IRAK4 as described in the Methods section. The proteins were used in their native form and were not chemically modified. An enzyme concentration-dependent increase in phosphorylation correlated with increasing fluorescence signal, demonstrating the efficacy of the QTL Lightspeed™ platform for detection of phosphorylated proteins (Figure 2). The detection of MBP phosphorylation worked equally well for protein substrates derived from either bovine or human (not shown). In order to explore the utility of the assay for screening inhibitors, the ATP competitor Staurosporine was used to inhibit enzyme activities using substrates Histone H1, PHAS-1 (Figure 3A) MBP (Figure 3B). For each protein, a concentration of Staurosporine which inhibited enzyme activity by 50% (IC_50_) was determined to be within the range of the reported value using a peptide substrate (9 nM) [17]: values of 3.8 nM, 1.6 nM and 0.6 nM were obtained for MBP, Histone H1 and PHAS-1, respectively (Figure 3). The IRAK4 protein assay was 3-fold more sensitive than a QTL Lightspeed™ assay performed on a peptide substrate (EC_50 _= 1.4 nM vs 5 nM). Using MBP as substrate, we were able to obtain IC_50 _values for Staurosporine of 11.5 nM, which were very similar to those obtained using a peptide substrate (19 nM, not shown).

The robustness of the assay (Z') was determined by performing 10 multiples of phosphorylation reactions using identical assay conditions. The Z' is a statistical parameter used in the drug screening community to evaluate and validate performance of assays [18]. An assay that delivers a Z' of higher than 0.5 is considered to be robust. The Z' was determined using the following equation:



For MBP, high Z' values of 0.84 and 0.8 were obtained for phosphorylation using PKCα and IRAK4, respectively. For PHAS-1 the values were 0.52 and for Histone H1 0.43. Table 1 summarizes the statistical values obtained for the three different substrates.

## Discussion

Despite the existence of several different technologies to monitor phosphorylation reactions of kinases, the detection of protein substrate phosphorylation has been limited to antibody-based assays, radioactive assays or assays that measure ATP consumption rather than specific kinase activity. Antibody-based assays are restricted to available, purified antibodies, usually directed against phosphoserine and phosphothreonine residues and may have low affinity and be specific to only a single kinase. Due to safety concerns over radioisotope handling as well as cost, drug screening efforts are rapidly moving toward non-radioactive and non-antibody-based assays. FP fulfills these criteria. However, since FP signal is correlated with changes in molecular rotation rates within the fluorescence life time and since only molecules smaller than approximately 20 kDa show significant rotation within this range most protein substrates are not directly measurable by FP [19]. A prompt fluorescence quench assay relies upon the binding of a phosphate coordinating metal ion to the phosphate group on a substrate, which is labeled with a dye [10]. Upon association of the metal ion, fluorescence is quenched. Similar to FRET assays, the rapid drop in signal with increasing distance between donor and acceptor molecules makes such assays unsuitable for the detection of protein phosphorylation, even if the proteins could be labeled with appropriate dyes [9]. The QTL Lightspeed™ protein assay is a novel non-radioactive and non-antibody-based assay that follows a simple mix and measure protocol for the quantitative detection of protein phosphorylation. Unmodified proteins can be used as substrates, thus allowing for the integrity of protein structure, which may be a requirement for successful recognition and phosphorylation by kinases. Our experiments utilized 0.5 μg MBP for phosphorylation by PKCα and IRAK4, thus moving the assay into the realm of HTS with regard to cost effectiveness. In this report, we describe the use of relatively small proteins MBP and PHAS-1 (18.9 kDa and 21 kDa respectively) and show feasibility for the larger protein Histone H1 (32 kDa) as well. Since our assay does not rely on site-specific antibodies, the phosphorylation of serine, threonine and tyrosine residues is possible.

## Conclusion

We demonstrate that a modified version of our QTL Lightspeed™ platform provides a simple and robust assay for monitoring protein phosphorylation. The platform does not employ radioactive labels or antibodies, which makes it specific as well as time and cost-effective. We show that the assays for PKCα are suitable for drug compound screening by obtaining IC_50 _values of an ATP-analog which are very close to those reported in the literature for this compound. For IRAK4, reported values were not available. Thus, it is reasonable that our assay is also well-suited for the identification of inhibitors that are not ATP-site directed competitors. Further applications of the assay could be the detection of autophosphorylated kinases or other proteins that play a role in cell signaling. Finally, the QTL Lightspeed™ platform can be used to further identify novel protein substrates for specific kinases.

## Methods

Fluorescent poly-phenylene ethyneylene was synthesized and coated onto microspheres as previously described [13,14].

Bovine substrate MBP was purchased from Upstate (Charlottesville, VA). PHAS-1 was purchased from Biomol (Plymouth Meeting, PA) and Histone H1 from Upstate. The phosphopeptide tracer was a 13 amino acid peptide, which was N-terminal labeled with a rhodamine derivative. The enzyme PKCα was purchased form Biomol and IRAK4 from Upstate. The inhibitor Staurosporine was from Sigma (St Louis, MO).

Kinase assays were performed using 384-well, white Optiplates (Perkin Elmer, Wellsley, MA) in a total volume of 15 μL. Protein Kinase Cα assays were performed in assay buffer (20 mM HEPES pH 7.4; 5 mM MgCl_2_; 0.1 mM CaCl_2_, 0.1 mg/ml 1-2-Dioleoyl-*sn *glycerol (Avanti, Alabaster, AL) and 0.02 mg/ml phosphatidylserine (Avanti) and 0.1% w/v NaN_3_) using 0.5 μg MBP as substrate for 1 hour at room temperature. The concentration of ATP (IDC, Livermore, CA) was 10 μM. IRAK4 phosphorylation was performed in assay buffer containing 50 mM Tris, pH 7.4; 5 mM MgCl_2_; 1 mM MnCl_2_; 0.1% BSA and 0.09% NaN_3_. The concentration of ATP was 50 μM for enzyme concentration curves and 10 μM for inhibition experiments. Following the enzymatic reaction, the phosphorylated protein was mixed with 15 μL of QTL Sensor for 10 minutes. Lastly, a rhodamine-labeled phosphorylated "tracer" peptide was added with a final concentration of 0.5 μM or 125 nM for detection of MBP or Histone H1 and PHAS-1 respectively for 30 minutes at ~25°C. The fluorescence of the reaction mixture was measured using a SpectraMax Gemini XS plate reader (Molecular Devices, Inc., Sunnyvale, CA) in well scan mode using excitation wavelength of 450 nm with a 475 nm cutoff filter and emission readout at 490 nm. Curve fitting was performed using GraphPad Prism^® ^sigmoid dose-response (variable slope) software.

## List of abbreviations

EC_50 _= enzyme concentration at 50% of signal; ELISA = Enzyme linked immunosorbent assay; FP = Fluorescence polarization; FRET = fluorescence resonance energy transfer; IC_50 _= Inhibitor concentration that produces 50% of signal.

## Authors' contributions

FR developed the experimental approach and wrote the manuscript; CS and WW carried out the experiments and DM made contributions to the conception of the study.

## Figures and Tables

**Table 1:** QTL Lightspeed™ Protein Assay Statistical Data^18^. Statistics are shown for Z' Factor, signal/noise (S/N), signal/background (S/B), signal window (SW) and coefficient of variation (% CV) at maximal substrate phosphorylation. Z'Factor equal to or greater than 0.5 are an indication of a robust assay^18^. Ten replicate measurements were made to determine the assay statistics.

Protein	Enzyme	Z' Factor	S/N	S/B	SW	%CV
MBP	PKCα	0.84	20.7	2.6	33.4	2.2
MBP	IRAK4	0.80	23	2.6	31	4.3
PHAS-1	PKCα	0.52	9.6	0.7	9.6	2.8
Histone H1	PKCα	0.43	7.32	0.7	9.4	3.0
